# Posture Detection of Dual-Hemisphere Capsule Robot Based on Magnetic Tracking Effects and ORB-AEKF Algorithm

**DOI:** 10.3390/mi16040485

**Published:** 2025-04-20

**Authors:** Xu Liu, Yongshun Zhang, Qiancheng Wang

**Affiliations:** State Key Laboratory of High-Performance Precision Manufacturing, Dalian University of Technology, Dalian 116024, China; zyshun@dlut.edu.cn (Y.Z.); wangqiancheng@mail.dlut.edu.cn (Q.W.)

**Keywords:** dual-hemisphere capsule robot, tracking effect, posture detection, ORB-AEKF algorithm

## Abstract

Posture detection is essential for capsule robots to be manipulated in a relatively closed gastrointestinal (GI) tract and to fulfill some medical operations. In this paper, a posture detection technique for a magnetic-actuated dual-hemisphere capsule robot (DHCR) is proposed. In this method, the DHCR realizes fixed-point posture adjustment based on tracking effects, and feature points are recognized and matched with the help of the ORB algorithm on the GI image acquired by a vision sensor. The system model is derived from the dynamic model and feature point information. Then, the posture is optimized by using the adaptive extended Kalman filter (AEKF) algorithm. As a result, the posture detection method based on the tracking effects and the ORB-AEKF algorithm is formed. The effectiveness and superiority of the proposed method are verified through experiments, which provide a good foundation for the subsequent, accurate closed-loop control of the DHCR.

## 1. Introduction

Magnetic-actuated capsule robots (MACRs) hold great potential for medical applications in the gastrointestinal (GI) tract [1,2]. Magnetic actuation is considered one of the most effective strategies because it allows for remote manipulation with high controllability, and it is more biocompatible and can penetrate human tissues without causing harm [3,4]. However, the individual variability in the GI tract tissue in terms of size, shape, and location, coupled with its relatively closed character, undoubtedly increases the difficulty of navigating MACRs in the GI tract. The actuation mechanism and posture estimation of MACRs play an important role in navigation maneuvers in the GI tract.

In general, the types of magnetic actuation can be divided into the magnetic traction type, which uses external permanent magnets [5] or electromagnets [6]; the magnetic force–torque hybrid type [7]; and the pure magnetic torque type, which uses paired coils [8]. The mechanisms of the first two types rely on static magnetic fields to establish a static equilibrium. However, this approach suffers from the coupling of magnetic force and torque, making it susceptible to disturbances that can affect the posture control of MACRs. In contrast, the mechanism of the third type employs dynamic magnetic fields to create a dynamic equilibrium. Based on this, a dual-hemisphere capsule robot (DHCR) system is proposed [9,10], which is actuated by a spatial universal rotating magnetic field (SURMF) generated by three-axis orthogonal square Helmholtz coils (TOSHCs). The DHCR exhibits a tracking effect [11] with good anti-interference capability and stability. This approach significantly enhances the accuracy of posture control compared to a static approach. However, the complex GI environment, the orientation error of the SURMF, and the dynamic response of the DHCR may all affect the actual axial orientation. Thus, realizing high-precision posture estimation remains a key challenge for clinical applications.

Currently, researchers are conducting in-depth studies on the posture detection of MACRs in the GI tract using different sensors and localization techniques [12], such as radio frequency, magnetic, inertial, medical imaging-based, and visual localization. Radio frequency can be used as a means of measurement using electromagnetic waves. Ye [13] used a radio frequency system for capsule localization. The advantage of this system is that it only requires a reading device to be installed outside the human body, allowing for remote measurements and an easy manipulation process. The human body is a heterogeneous and highly attenuating environment for radio frequency signals, which affects the quality of the read signal and reduces localization accuracy. Inertial detection technology relies on inertial measuring units such as gyroscopes and accelerometers. It has the advantages of autonomous observation and continuous dynamic localization. Vedaei [14] embedded inertial measuring units into a capsule to achieve the feedback of motion data. However, the inertial detection error may grow over time when performing continuous dynamic measurements. Thus, inertial detection techniques are rarely used alone and usually need to be combined with other techniques in practical applications. Magnetic localization utilizes sensors to detect magnetic sources, and its methods can be classified into two types. For example, Xu [15] employed an external magnetic sensor array for the pose parameters of MACRs, which is a passive method; Popek [16] proposed a method for estimating the posture of MACRs with embedded Hall effect sensors, using a rotating dipole field, which is an active method. The external sensor arrangement in the first method can be limited by the working space. Built-in sensors in the second method consume more limited on-board space and power. Meanwhile, the magnetic sensors may experience interference with the magnetic field used to drive the MACRs, affecting detection accuracy. Medical imaging-based localization techniques mainly include the use of X-ray, computed tomography, and magnetic resonance imaging. Marya [17] used this technique to image MACRs, providing data to support the assessment of the accuracy of the localization technique. It should be noted that the human body should not be irradiated frequently or for long periods of time to avoid radiation damage. Visual localization [18] uses a video of the GI tract captured by MACRs as the localization source. Because there are no internal or external sensor devices, MACRs save internal space. In addition, endoscopists in general also clarify the subsequent operations to be performed based on the GI video to realize the complete navigation task. Although this method enables visualization, the detection of pose accuracy in the complex GI tract environment remains a challenge. There are also several localization methods that have been heavily researched, such as the ultrasonic [19], the deep learning and neural network-based [20,21], and the multi-sensor fusion localization methods [22]. For the DHCR proposed in this study, no gyroscope sensors or Hall sensors are installed inside the capsule, considering the size and energy constraints, so vision technology is utilized to achieve posture detection.

In practical applications, factors such as sensor noise, nonlinear motion, and external interference can affect the accuracy of posture estimation. The dynamic response characteristics of the DHCR during posture adjustment are also not taken into account, resulting in large errors in some of the detected values [23]. Extended Kalman filtering (EKF) with the covariance matching method is denoted as adaptive extended Kalman filtering (AEKF), which can be used to solve this problem. Therefore, to improve the posture detection accuracy, this paper proposes a DHCR posture detection method based on the ORB-AEKF algorithm with the aid of the dynamic motion model. This method fuses magnetic control and visual localization techniques without additional sensors and provides the key support for the closed-loop control of DHCR.

This paper is organized as follows: Section 2 introduces the DHCR posture detection method. Section 3 describes the tracking effects mechanism and establishes the motion model. Section 4 elaborates on the ORB-AEKF algorithm. Section 5 experimentally validates the posture detection method. Finally, Section 6 gives a conclusion.

## 2. Posture Detection Method Overview

### 2.1. Magnetic-Actuated DHCR Diagnostic System

**Figure 1:**
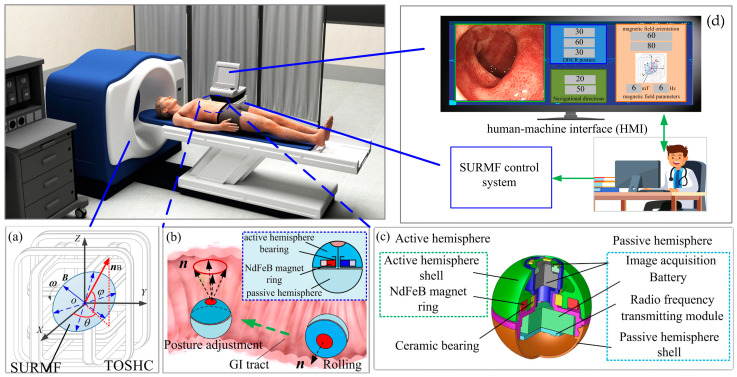
Magnetic-actuated DHCR diagnostic system. (**a**) TOSHC; (**b**) schematic diagram; (**c**) 3D design of DHCR; (**d**) HMI communication control system.

The magnetic-actuated DHCR diagnostic system consists of a SURMF generating device, a DHCR, and a human–machine interaction (HMI) communication control system, as shown in Figure 1.

The SURMF generating device (TOSHC, as shown in Figure 1a) produces a SURMF when a three-phase alternating current is applied to the coils. The specific current formula is shown in [10]. The SURMF with magnetic vector ***B*** rotates counterclockwise with angular velocity ***ω*** around the axis normal vector ***n****_B_*= (*θ*, *ϕ*), where *θ* and *ϕ* are the yaw and the pitch angles of ***n****_B_*, respectively.

**Figure 2:**
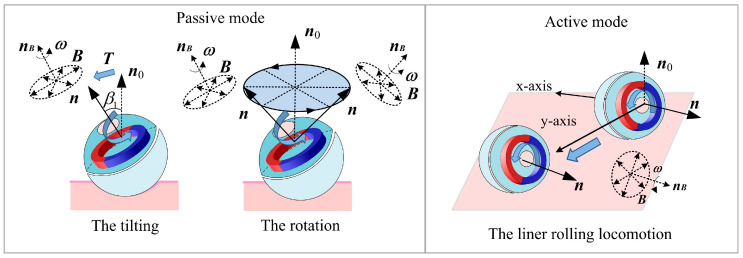
Passive and active modes of DHCR.

The spherical DHCR comprises active and passive hemispheres, as shown in Figure 1b, connected by a ceramic bearing. The active hemisphere contains an NdFeB radial magnetizing ring actuator, whereas the passive hemisphere is underdriven. The magnetic dipole moment ***m*** and the magnetic vector ***B*** generate magnetic torque ***T*** rotating the active hemisphere via the magnetic ring. Based on tracking effects, the DHCR can align its axis with the applied magnetic field direction. As shown in Figure 2, two distinct working modes emerge based on DHCR-GI tract contact: (1) when only the passive hemisphere contacts the tract, the fixed-point posturing is performed in the passive mode; (2) when the active hemisphere contacts the GI tract, it is converted to the active mode, and rolling walking is performed. The passive hemisphere integrates an image acquisition module, wireless transmission module, and power supply for system communication, as shown in Figure 1c. 

The HMI communication control system mainly contains an HMI and a SURMF controller to receive and display GI tract images for endoscopic diagnosis, as shown in Figure 1d. Then, by sending control signals to the SURMF controller, operators can adjust the orientation, intensity, and rotation speed of the SURMF to guide the DHCR to complete clinical procedures.

### 2.2. Posture Detection Method

**Figure 3:**
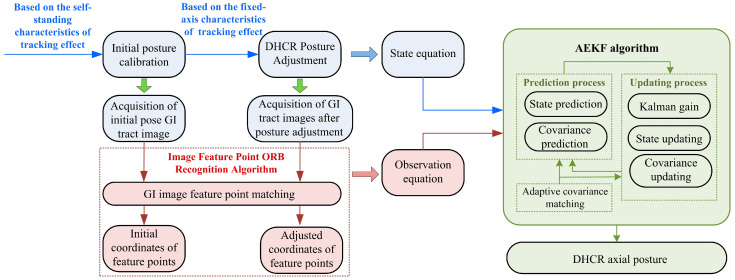
Posture detection method flowchart diagram.

The posture detection method consists of three main components: (1) DHCR posture calibration and adjustment based on tracking effects, (2) GI tract image acquisition by the vision system and feature points recognition based on the ORB algorithm, and (3) posture estimation based on the AEKF, as shown in Figure 3.

First, the DHCR initial posture calibration is performed. As the DHCR undergoes multimodal conversion in the GI tract, the starting posture calibration is realized based on the self-standing characteristic of tracking effects. This ensures that the DHCR aligns its axial in a vertical upward orientation. In addition, in the vertical orientation, the magnetic vector azimuthal difference is zero, which reduces the effect of phase difference caused by coil inductance. Therefore, the vertical orientation is taken as the initial posture, and the DHCR vision module captures GI tract images. Afterwards, when posture adjustment is performed based on the fixed-axis characteristic of tracking effects, the DHCR vision module is utilized to capture GI tract images during the process. During the movement of DHCR based on tracking effects, its axial posture is defined as the state vector, and the system state equation is constructed with the aid of the DHCR dynamics model.

Then, after capturing GI tract images before and after posturing, the improved ORB algorithm is utilized to recognize and match feature points. The matching relationship and pixel coordinates of the feature points are obtained. Since there are no other sensors inside the DHCR, the feature point pixel coordinates become the system observed values, and the observation equations are constructed based on the functional relationship between the image and world coordinate systems.

Finally, because the state and observation equations of the DHCR system are nonlinear, the AEKF is used for posture estimation. After linearizing the state and observation equations, the current state estimation value (DHCR axis orientation) is iteratively updated using prior state estimates and current observations. The AEKF algorithm considers visual detection errors, effectively integrates prediction and observation information, and ultimately optimizes state estimation.

In summary, the above progress constitutes a DHCR posture detection method based on the ORB-AEKF algorithm. This method incorporates the DHCR system motion characteristics on the basis of visual detection, improving the posture detection effect.

## 3. Tracking Effects Mechanism and Motion Model Establishment

### 3.1. Tracking Effects Analysis

**Figure 4:**
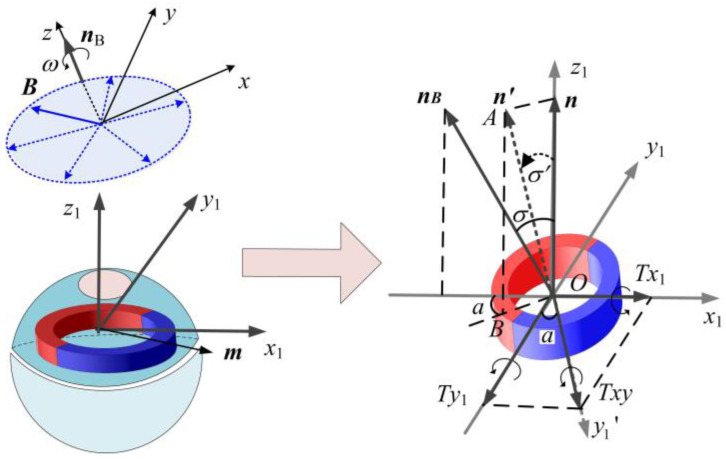
Torque on the magnetic ring during deflection motion.

After placing the DHCR in the SURMF, the DHCR is guided to adjust its posture by controlling the SURMF, achieving multimodal motion. Assuming that the angle between the SURMF orientation ***n****_B_* and the magnetic ring orientation ***n*** is *σ*, a coordinate system *O*-*x*_1_*y*_1_*z*_1_ is introduced, where axis *Oy*_1_ aligns with the line orthogonal to both ***n*** and ***n***_B_, and the axis *Oz*_1_ coincides with axis ***n***, as shown in Figure 4.

The rotating magnetic vector ***B*** can be represented in the *O*-*x*_1_*y*_1_*z*_1_ coordinate system as follows:

(1) $$B_{2}=\left(\begin{array}{ccc} \cos\sigma & 0 & -\sin\sigma \\ 0 & 1 & 0\\ \sin\sigma & 0 & \cos\sigma \end{array}\right)\left(\begin{array}{c} B_{0}\cos\omega t\\ B_{0}\sin\omega t\\ 0 \end{array}\right)=\left(\begin{array}{c} B_{0}\cos\sigma \cos\omega t\\ B_{0}\sin\omega t\\ B_{0}\sin\sigma \cos\omega t \end{array}\right)$$

where *B*_0_ is the magnetic flux density. A previous study [11] showed that the magnetic ring synchronously rotates with ***B*** but lags by a hysteresis angle *δ*. Thus, the magnetic dipole moment ***m*** can be expressed in the *O*-*x*_1_*y*_1_*z*_1_ coordinate system as follows:

(2) $$m={\left(\begin{array}{ccc} m_{0}\cos(\omega t-\delta ) & m_{0}\sin(\omega t-\delta ) & 0 \end{array}\right)}^{T}$$

where *m*_0_ is the modulus of the magnetic moment and *δ* is the slip angle.

The coupling magnetic torque ***T*** can be expressed in the *O*-*x*_1_*y*_1_*z*_1_ coordinate system as follows:

(3) $$T=m\times B_{2}={\left(\begin{array}{ccc} T_{x1} & T_{y1} & T_{z1} \end{array}\right)}^{T}=m_{0}B_{0}\left(\begin{array}{c} \sin(\omega t-\delta )\cos\omega t\sin\sigma \\ -\cos(\omega t-\delta )\cos\omega t\sin\sigma \\ \cos(\omega t-\delta )\sin\omega t-\sin(\omega t-\delta )\cos\omega t\cos\sigma \end{array}\right)$$

The magnetic torque component ***T****_z_*_1_ drives ***m*** to rotate around the axis *Oz*_1_, while *T_y_*_1_ and *T_x_*_1_ drive the magnetic ring to deflect around the axis *Oy*_1_ and *Ox*_1_, respectively. Although *T_x_*_1_ and *T_y_*_1_ are time-varying, their periodic nature causes oscillations around an equilibrium point with a period of *τ*′ = π /*ω*. By applying the period-averaging method, the equivalent magnetic torque components ${\bar{T}}_{x1}$ and ${\bar{T}}_{y1}$ are derived as follows:

(4) $$\left(\begin{array}{c} {\bar{T}}_{x1}\\ {\bar{T}}_{y1} \end{array}\right)=m_{0}B_{0}\left(\begin{array}{c} \frac{1}{\tau '}\int_{0}^{\tau '}T_{x1}dt\\ \frac{1}{\tau '}\int_{0}^{\tau '}T_{y1}dt \end{array}\right)=\frac{m_{0}B_{0}}{2}\left(\begin{array}{c} \sin\sigma \sin\delta \\ -\sin\sigma \cos\delta \end{array}\right)$$

Then, the equivalent total deflection torque ${\bar{T}}_{xy}$ and its equivalent azimuth $\bar{a}$ are derived as follows:

(5) $$\begin{array}{l} {\bar{T}}_{xy}=-\sqrt{{\bar{T}}_{x1}^{2}+{\bar{T}}_{y1}^{2}}=-\frac{B_{0}m_{0}}{2}\sin\sigma \\ \bar{a}=\arctan\frac{T_{x1}}{T_{y1}}=\arctan(\tan\delta )=\delta \end{array}$$

**Figure 5:**
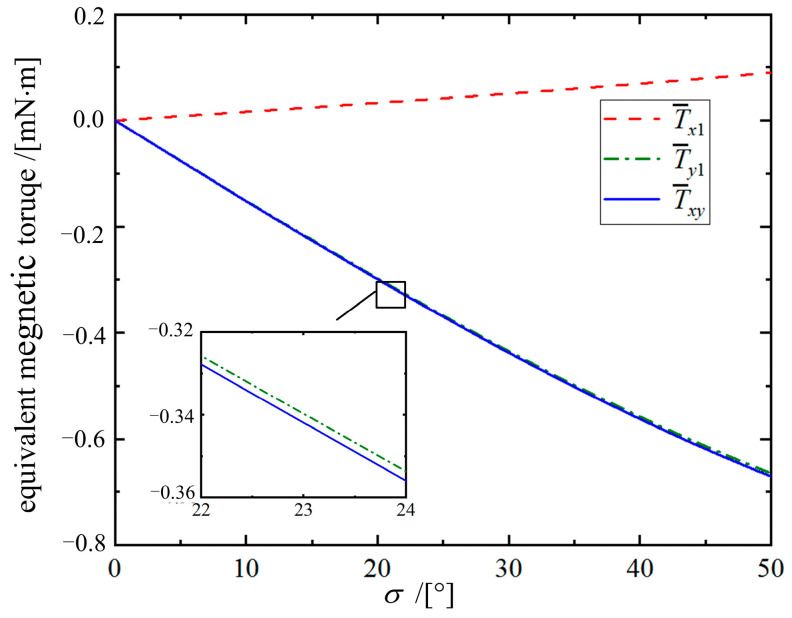
Curves of each equivalent magnetic torque versus angle *σ*.

The relationship curves between the equivalent magnetic torques and angle *σ* are shown in Figure 5. It can be seen that ${\bar{T}}_{xy}$ is basically close to ${\bar{T}}_{y1}$, which indicates that ${\bar{T}}_{y1}$ dominates the deflection motion so that ***n*** always tends to deflect toward ***n***_B_. However, when *σ* is not 0°, ${\bar{T}}_{x1}$ always exists. Therefore, the magnetic ring deflects around the *Oy*_1_′ axis within the *OAB* plane, rather than directly around the *Oy*_1_ axis within the *Ox*_1_*z*_1_ plane. The angle between the *Ox*_1_*z*_1_ plane and the *OAB* plane is $\bar{a}$, representing the deviation degree of ***n*** during motion. 

**Figure 6:**
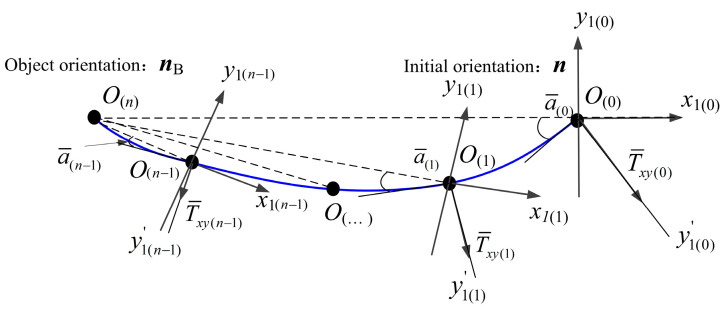
Schematic of the magnetic ring axis end trajectory.

Figure 6 illustrates the magnetic ring axis trajectory. As *σ* gradually approaches 0°, ${\bar{T}}_{xy}$ decreases to 0 mN⋅m, causing ***n*** to align with ***n***_B_. At this equilibrium state, since the DHCR is coaxial with the magnetic ring, its axis remains fixed. It indicates that after applying SURMF in any direction, the axis ***n*** of the DHCR can always track the axis ***n***_B_ and ultimately maintain coaxial, forming tracking effects. The fixed-axis characteristic serves as the fundamental mechanism for DHCR to achieve fixed-point posture adjustment. 

When a vertically oriented magnetic field is applied, the DHCR axis rapidly aligns with the vertical direction, achieving a self-standing state. The whole process demonstrates the unique self-standing characteristic of DHCR [10]. 

### 3.2. Motion Model Establishment

Posture detection in passive mode requires model-aided techniques, necessitating a clear dynamic model of the DHCR. The DHCR is a multi-rigid body structure with internal forces. To avoid solving internal forces, the Lagrangian method is used to construct the dynamic equations. The specific modeling process is shown in Appendix A. Eventually, the dynamic equation can be obtained as follows:

(6) $$M(q)\ddot{q}+C(q,\dot{q})\dot{q}+M_{f}(\dot{q})=Τ$$

where ***q*** is Euler angles; ***T*** is the magnetic torque; $M_{f}(\dot{q})$ is the resistance torque; and M(q) and $C(q,\dot{q})$ are the inertia matrix, and the centrifugal force and Coriolis force matrix, respectively.

To avoid singularities inherent in the Euler or Cardan angle representations, the DHCR posture is described by quaternions ***p*** (in Appendix B). The conversion between Euler angles (*α* and *β*) and quaternions is given by the following:

(7) $$\begin{array}{l} \alpha =\arctan\frac{2(p_{0}p_{1}+p_{2}p_{3})}{1-2({p_{1}}^{2}+{p_{2}}^{2})}\\ \beta =\arcsin(2(p_{0}p_{2}-p_{3}p_{1})) \end{array}$$

where *p_i_* (*i* = 0, 1, 2, 3) is the Rodriguez parameter.

## 4. Posture Detector Design Based on ORB-AEKF Algorithm

The posture detection method consists of a quaternion-based state equation, an observation equation based on the ORB algorithm, and posture estimation based on the AEKF algorithm.

### 4.1. State Equation

The DHCR axis posture is expressed based on quaternions as the state vector. The state vector and its time derivative can be expressed as follows:

(8) $$\begin{array}{l} X_{t}={\left(\begin{array}{cccc} p_{0} & p_{1} & p_{2} & p_{3} \end{array}\right)}^{T}\\ {\dot{X}}_{t}=\frac{1}{2}\Omega (\omega )X_{t} \end{array}$$

where the angular velocity matrix ***Ω***(*ω*) is defined in Appendix B.

Applying the first-order Runge–Kutta method to solve the differential equation yields the following discrete form of the state equation:

(9) $$X(k+1)=f[k,X(k)]=[I_{4}+\frac{1}{2}\Omega (\omega )\Delta k]X(k)$$

Considering the model uncertainties and dynamic noise ***W***, Equation (9) can be modified as follows:

(10) X(k+1)=f[X(k)]+W(k)

where the dynamic noise ***W***(*k*) is Gaussian white noise with mean zero and covariance matrix ***Q***(*k*).

### 4.2. Observation Equation

#### 4.2.1. GI Image Feature Point Recognition and Matching Based on the ORB Algorithm

**Figure 7:**
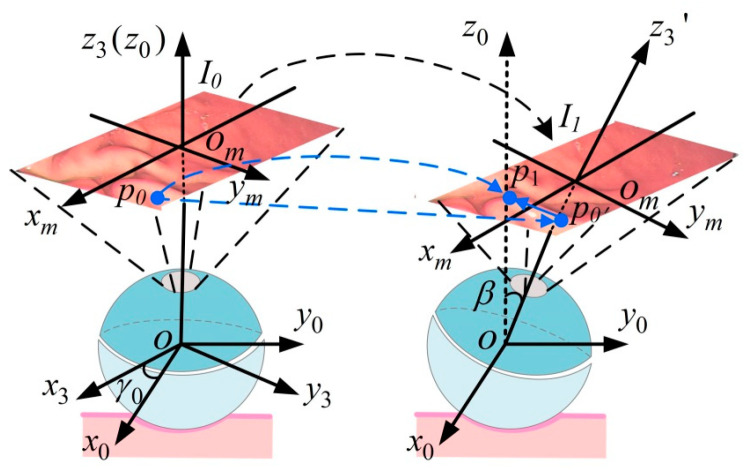
Acquisition of GI tract images before and after DHCR posture adjustment.

Due to the volume and energy limitations, the DHCR does not incorporate gyroscopic sensors. Instead, posture information can be detected through the vision system. During the posture adjustment process of DHCR based on tracking effects, GI images can be obtained, as shown in Figure 7.

For GI image processing, we evaluated both traditional (ORB algorithm) and deep learning approaches (self-supervised learning [24] and SuperPoint [25]). After comparison, it is found that although deep learning methods have become mainstream in machine vision, there are still some limitations in the application process. First, the complex GI environment requires extensive labeled training data, increasing preparation difficulty. Second, substantial computational demands reduce real-time performance, particularly problematic given the need for GPU acceleration in resource-constrained scenarios. In contrast, the ORB algorithm is a lightweight feature extraction algorithm with a processing time of about 378 ms and significantly lower resource requirements than deep learning schemes. The ORB algorithm has better robustness in processing GI tract images, smaller computation, and a better quasi-matching effect, which has been validated in a series of experiments in GI tract environments with different light and blurring conditions [26]. Therefore, this paper selects the ORB algorithm for GI tract feature matching to provide data support for the subsequent posture estimation.

**Figure 8:**
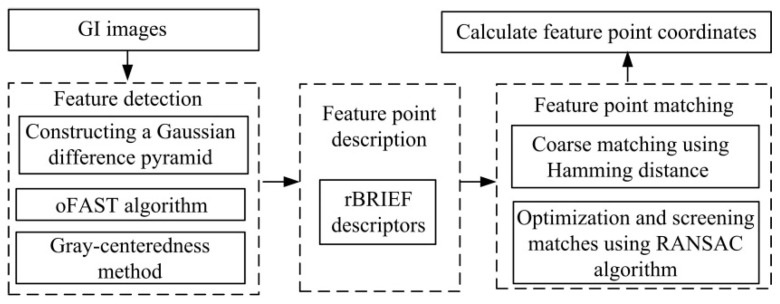
Flowchart of feature point recognition and matching based on the ORB algorithm.

**Figure 9:**
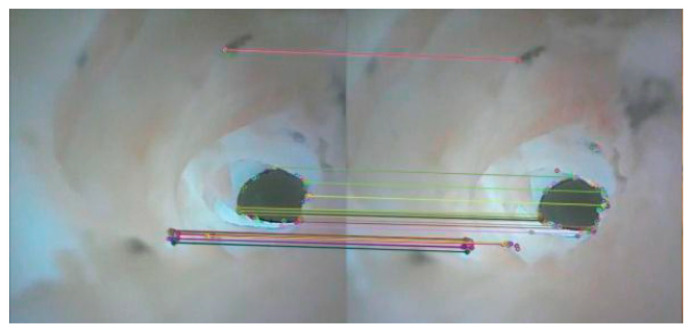
GI images feature points recognition and matching results, where different colored lines represent matching lines for different feature points.

As shown in Figure 8, the ORB algorithm divides the processing of image feature information into two parts: the feature detection and the feature point description, which consists of the improved oFAST corner point detection algorithm and the rBRIEF descriptor. After the image features are detected, feature matching is required to establish the relationship between the images, which is achieved by comparing the similarity relationship between the feature points. After the binary descriptors are obtained from the ORB algorithm, Hamming distance is used to match feature points coarsely. In order to reduce the matching error, outlier feature matches are removed using a random sampling method. The processed GI images yield the feature matching results shown in Figure 9, from which we extract the precise feature point coordinates for subsequent posture estimation.

#### 4.2.2. Observation Equation Establishment

The system observation vectors are the feature point pixel coordinates in the two-dimensional pixel coordinate system, which maintain a specific constraint relationship with the state vectors. To establish this relationship, we define the following coordinate systems.

**Figure 10:**
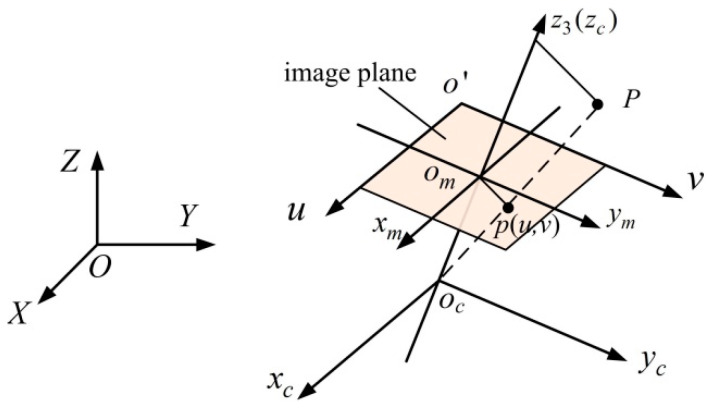
Camera linear imaging model of coordinate transformation relationships.

The world coordinate system *O*-*XYZ*, camera coordinate system *o_c_-x_c_y_c_z_c_*, image physical coordinate system *o_m_-x_m_y_m_*, and image pixel coordinate system *o*′-*uv* are introduced, as shown in Figure 10. The camera is solidly connected to the passive hemisphere, and *o_c_-x_c_y_c_z_c_* is established with the center *o_c_* of the camera as the origin, in which the optical axis is the *o_c_z_c_* axis. In *o_m_-x_m_y_m_*, the plane *x_m_o_m_y_m_* is perpendicular to the *o_c_z_c_* axis, and the intersection point is *o_m_*. The *o_m_x_m_* and *o_m_y_m_* axes parallel the *o_c_x_c_* and *o_c_y_c_* axes, respectively. The camera focal length is *o_c_o_m_*, which is usually denoted by *f*. To describe the image pixel point location, the image pixel coordinate system *o*′-*uv* is constructed, where the coordinate system origin is located in the upper left corner of the image plane, and the *o*′*u* and *o*′*v* axes are parallel to the *o_m_x_m_* and *o_m_y_m_* axes, respectively.

In the passive mode, the DHCR vision module remains fixed to the passive hemisphere, causing the camera pose to be equivalent to directly present the DHCR posture. Leveraging the isotropic homogeneity of the SURMF, the origin of the camera coordinate system can coincide with the origin of the world coordinate system. Then, *o_c_-x_c_y_c_z_c_* completes the coordinate conversion to *O*-*XYZ* through the rotation matrix ***R***. Take a point *P* in space as an example to describe the mapping relationship from *O*-*XYZ* to *o*′-*uv*. For a spatial point *P*(X, Y, Z) in *O*-*XYZ*, with project image point *p* (*u*, *v*) in *o*′-*uv*, the transformation from *O*-*XYZ* to *o*′-*uv* can be expressed as follows:

(11) $$z_{c}\left(\begin{array}{c} u\\ v\\ 1 \end{array}\right)=\left(\begin{array}{cc} K & 0 \end{array}\right)\left(\begin{array}{cc} R_{I} & 0\\ 0 & 1 \end{array}\right)\left(\begin{array}{c} X\\ Y\\ Z\\ 1 \end{array}\right)=\left(\begin{array}{cccc} f/dx & 0 & u_{0} & 0\\ 0 & f/dy & v_{0} & 0\\ 0 & 0 & 1 & 0 \end{array}\right)\left(\begin{array}{cc} R_{I} & 0\\ 0 & 1 \end{array}\right)\left(\begin{array}{c} X\\ Y\\ Z\\ 1 \end{array}\right)$$

where ***K*** is the camera internal reference matrix; ***R****_I_* is the rotation matrix; d*x* and d*y* are the pixel physical dimensions; (*u*_0_, *v*_0_) is the point where the imaging plane intersects the optical axis, which is also called the main point and is theoretically located at the geometric center of the image; and *z_c_* is the *o_c_z_c_* axis coordinate of *P* in *o_c_-x_c_y_c_z_c_*.

When the initial posture calibration of DHCR is performed based on tracking effects, ***R*** is **0**. Assuming that the feature point is initially in *o*′-*uv* with the coordinates (*u_s_*, *v_s_*), according to Equation (11), the coordinates of the feature point *P_s_* can be calculated as follows:

(12) $$P_{s}=\left(\begin{array}{c} X_{s}\\ Y_{s}\\ Z_{s} \end{array}\right)=\left(\begin{array}{c} z_{s}(u_{s}-u_{0})dx/f\\ z_{s}(v_{s}-v_{0})dy/f\\ z_{s} \end{array}\right)$$

Assuming that the pixel coordinate of the feature point obtained at moment *k* is (*u_k_*, *v_k_*), according to Equation (11), the following is obtained:

(13) $$\left(\begin{array}{c} u_{k}\\ v_{k} \end{array}\right)=\frac{F_{c}\cdot R_{Ik}\cdot P_{s}}{E_{1}\cdot R_{Ik}\cdot P_{s}}$$

where

(14) $$F_{c}=\left(\begin{array}{ccc} f/dx & 0 & u_{0}\\ 0 & f/dy & v_{0} \end{array}\right),E_{1}=\left(\begin{array}{ccc} 0 & 0 & 1 \end{array}\right)$$

and ***R****_Ik_* derives from Equation (A6).

Based on Equation (13), the observation equation of the system is expressed as follows:

(15) Z(k)=h[k,X(k)]

Clinical preparations (8-h fasting and consuming simethicone) improve the GI visualization [27], but illumination variations and motion blur persist, introducing observation noise ***V***. The observation equation can be modified as follows:

(16) Z(k)=h[k,X(k)]+V(k)

where the observation noise ***V***(*k*) is Gaussian white noise with mean zero and covariance matrix ***R***(*k*), calibrated per [26].

### 4.3. AEKF Algorithm

Posture estimation optimization is achieved by solving the posterior probability distribution of the state quantities. Methods such as the Kalman filter, EKF, unscented Kalman filter, and particle filter are usually used [28]. Since the DHCR dynamic system and the observation equations are nonlinear, the standard Kalman filter is inapplicable. The unscented Kalman filter can capture higher-order nonlinearities and is suitable for high-accuracy applications in controlled environments, but it is sensitive to uncertain noise. The particle filter gives more accurate results with sufficient samples but is computationally intensive and also faces sample impoverishment. In contrast, EKF is suitable for scenarios with uncertain noise, while the computational complexity is relatively small, and is the most widely used nonlinear state estimation technique. Therefore, EKF is selected for the posture estimation of DHCR in this paper.

The EKF linearizes the nonlinear system, involving Equations (10) and (16), through first-order Taylor expansion, enabling the application of the Kalman filter algorithm. An accurate a priori covariance matrix plays a key role in applying EKF for posture estimation, which involves the process noise covariance matrix ***Q*** and the observation noise covariance matrix ***R***. The inaccurate noise variance estimation can lead to a decrease in estimation accuracy. The essence of the adaptive method is to correct the noise variance in real time, by automatically updating the noise covariance matrix in the algorithm to make it closer to the real characteristics of the noise. In this paper, the covariance matching method [29] is introduced to realize the noise adaptive.

The AEKF algorithm executes through three sequential phases: prediction, updating, and adaptive variance matching. In the prediction phase, the system state equations, observation equations, and error variance matrix are predicted. In the updating phase, the state prediction equation is modified, and the state error covariance matrix is updated based on the calculated Kalman gain. Finally, the adaptive variance matching of observation and process noise is realized by introducing scale factors to update the Kalman gain and prediction error variance matrices. The specific process is depicted in Appendix C.

**Figure 11:**
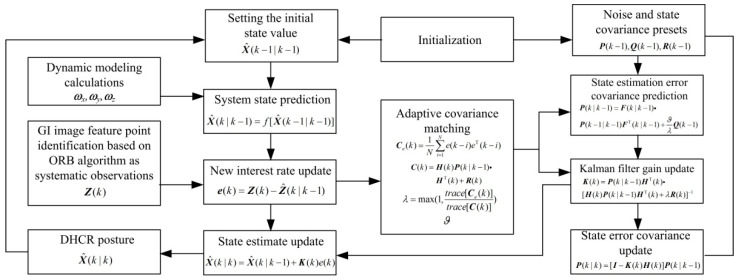
Calculation flowchart of DHCR posture detection based on ORB-AEKF algorithm.

In summary, combined with the tracking effects motion mechanism, the DHCR detection method based on ORB-AEKF is formed. The specific algorithm flow block diagram is shown in Figure 11.

## 5. Experiment

### 5.1. Experimental Platform

**Figure 12:**
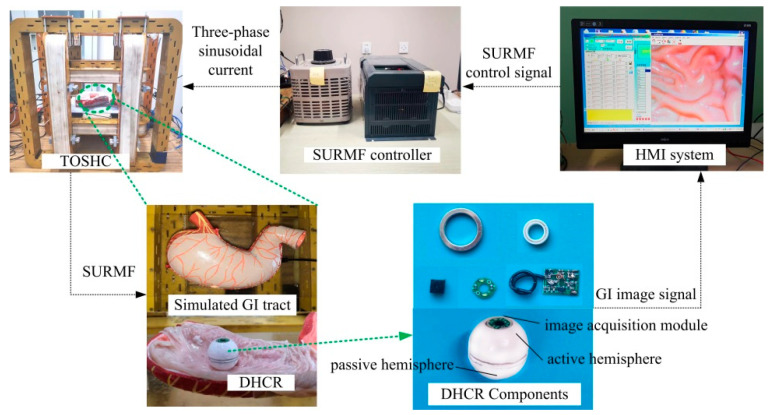
Experimental platform.

To verify the effectiveness of the posture estimation method proposed in this paper, an experimental platform is built, comprising a DHCR, a TOSHC, an HMI communication system, and a simulated GI tract, as shown in Figure 12. The DHCR prototype has been developed, integrating a radial magnetized NdFeB magnetic ring, miniature ceramic bearing, battery, image acquisition module, and radio frequency transmitting module to a sphere with a dual-hemisphere structure. During experiments, the DHCR is placed in the simulated GI tract. The simulated stomach model used is made of silicone, and it is approximately 25 cm in length, 10 cm in width, and 8 cm in height, with a lumen volume of 1 L. In addition, during the experiments, the surface of the stomach model is lined with isolated porcine colon to simulate real tissue. During operation, the DHCR transmits the GI tract image to the computer. After inputting parameters to the SURMF controller, the controller feeds three-phase currents into the TOSHC to generate SURMF. The DHCR can be actuated by SURMF based on tracking effects to realize multimodal motions.

The maximum working magnetic field strength of the TOSHC is maintained below 15 mT, with a maximum rotational frequency of 10 Hz. The magnetic field radiation and thermal effects meet the safety requirements [30,31].

### 5.2. Tracking Effects Verification Experiments 

**Figure 13:**
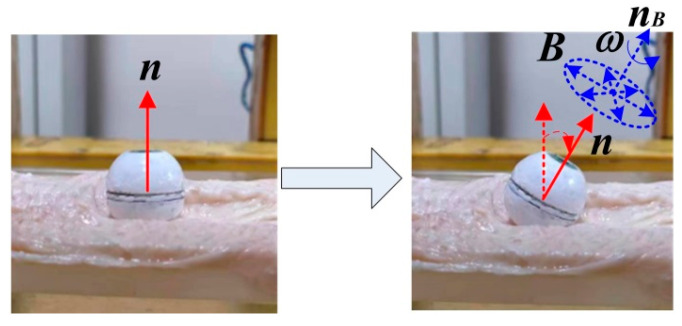
Tracking effect experiment of the DHCR.

Tracking effects is the core mechanism by which the DHCR performs fixed-point posturing. During experiments, when the SURMF with different axial orientations is applied, the DHCR axis orientation can be rapidly deflected from initial orientations to the set target orientation, as shown in Figure 13.

**Figure 14:**
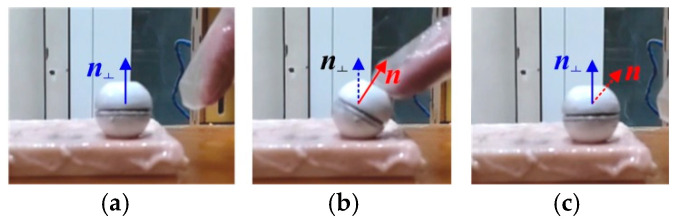
DHCR anti-interference experiment. (**a**) Initial state; (**b**) manual interference; (**c**) posture recovery.

Due to the complex GI environment, the DHCR may experience various disturbances. External disturbances are simulated through manual interference to test the immunity to interference in the fixed-axis characteristics of DHCR, as shown in Figure 14.

In the anti-interference experiment, the applied SURMF axis is vertically upward. The DHCR axis quickly aligns the vertical orientation after applying the magnetic field, as shown in Figure 14a. Physical disturbance is simulated through manual interference, causing axis deviation from vertical, as shown in Figure 14b. After disturbance cessation, the DHCR axis can quickly return to vertical orientation, as shown in Figure 14c. The experiments show that the DHCR exhibits excellent stability and anti-interference properties, enabling it to maintain posture stability against natural peristalsis and other GI tract disturbances while performing modal functions.

### 5.3. ORB-AEKF-Based Posture Detection Verification Experiments

#### 5.3.1. Simulated GI Condition Experiments

**Figure 15:**
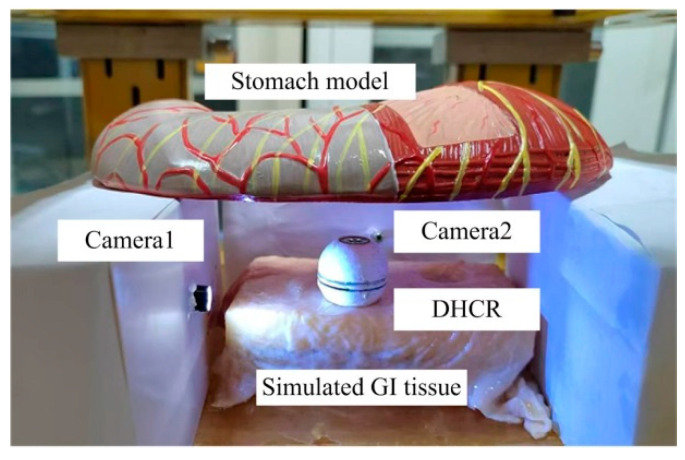
DHCR posture measurement experimental setup.

**Figure 16:**
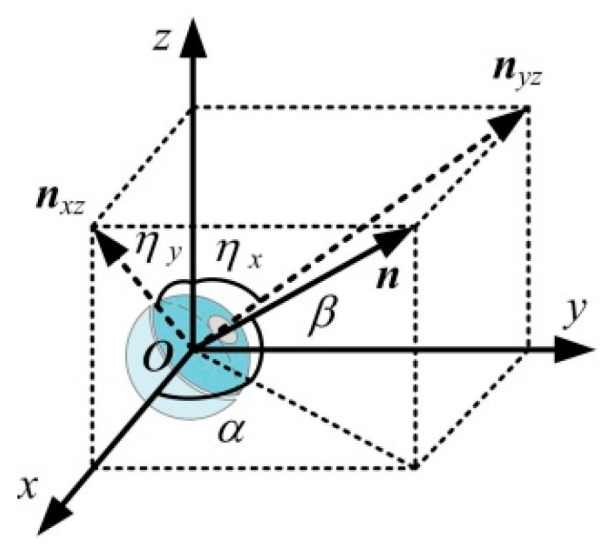
DHCR posture measurement principle.

To validate the DHCR posture detection method, a DHCR posture measurement experimental setup is constructed, as shown in Figure 15. Two cameras are set up along the *x*-axis and *y*-axis to observe and record the DHCR axis ***n*** during posture adjustment. The angles (*η_x_* and *η_y_*) between the axial projections *n_yz_* and *n_xz_* and the vertical *z*-axis are measured in each camera view, as shown in Figure 16. Based on the geometric relationship, the DHCR posture is calculated as follows:

(17) $$\left\{\begin{array}{l} \alpha =\arctan(\frac{\tan\eta_{x}}{\tan\eta_{y}})\\ \beta =\arctan(\frac{1}{\sqrt{\tan^{2}\eta_{x}+\tan^{2}\eta_{y}}}) \end{array}\right.$$

After averaging the above equation over several calculations, the posture angle obtained is used as a reference value to compare with the posture estimate. 

Prior to the experiments, camera calibration was performed using Zhang's method to obtain the camera internal reference matrix ***K***:

(18) $$K=\left(\begin{array}{ccc} 466.14 & 0 & 320\\ 0 & 466.14 & 240\\ 0 & 0 & 1 \end{array}\right)$$

**Figure 17:**
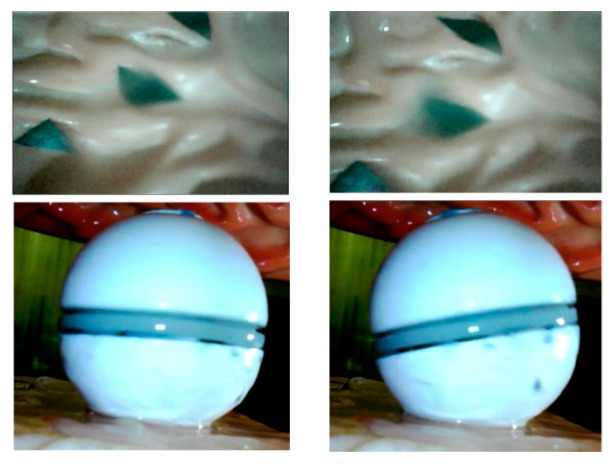
DHCR posture measurement process image.

The DHCR posture detection procedure comprised the following steps: (1) The DHCR is placed in the simulated GI tract, with verification of proper image acquisition from both the embedded DHCR camera and external Cameras 1–2. (2) The TOSHC generated a vertically upward SURMF. Based on the self-standing characteristic, the DHCR starting posture calibration is performed, and the corresponding GI image is recorded. (3) Multiple target magnetic field orientations are set. Based on the fixed-axis characteristic, the DHCR performs fixed-point alignment, with GI images recorded during adjustment process, as shown in Figure 17. (4) The ORB-AEKF algorithm processes the recorded images to extract the DHCR’s axis posture. Key calculation parameters are listed in Table 1.

**Figure 18:**
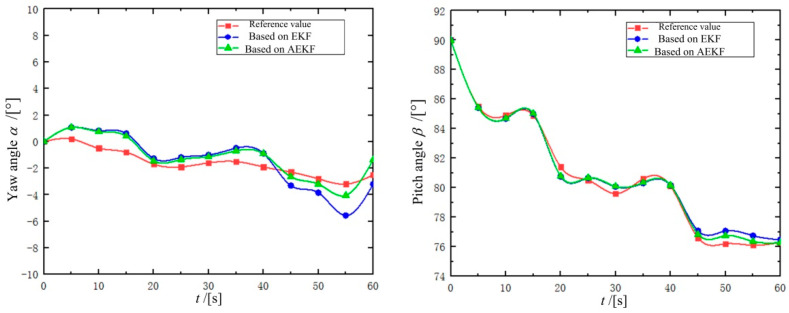
DHCR posture detection results.

To evaluate the robustness of the proposed posture detection method, we compared its performance with a conventional EKF-based approach that lacked adaptive covariance matching. Figure 18 presents the comparative results through plotted curves of the DHCR's axial yaw and pitch angles. The data reveal that while both methods successfully capture the general posture trends of the DHCR, their performance diverges with increasing deflection angles. During initial stages with minimal deflection, both methods produce nearly identical results. However, as the deflection angle grows, the ORB-AEKF-based method demonstrates superior accuracy, with its estimates aligning more closely with the reference values than those generated by the standard EKF approach. 

To quantitatively evaluate the detection accuracy of each posture detection method, we compared their performance using three key metrics: maximum error, mean error, and error variance. Table 2 presents these statistical measures for all tested methods, enabling direct comparison of their precision and stability.

Table 2 shows that the posture detection method based on ORB-AEKF achieves significant improvements across all error metrics, with reductions in maximum error, mean error, and variance compared to alternative methods. The average error values of yaw angle and pitch angle are 0.76° and 0.31°, respectively, and the variance is minimized, which indicates that the posture detection results are robust. The results prove the superior performance of the proposed posture detection method. The yaw angle detection error is slightly lower than the pitch angle detection error, most likely due to gravity center offset [32]. The gravity center offset torque becomes a dynamic disturbance torque in the yaw motion since the yaw magnetic torque and the gravity center offset torque are orthogonal vectors, which cause a larger detecting error in the yaw angle. Meanwhile, the gravity center offset torque and the pitch magnetic torque are isotropic, which will not interfere with the pitch motion, so the pitch angle detecting error is lower.

#### 5.3.2. Posture Detection Application to the Navigation in Porcine Colon

**Figure 19:**
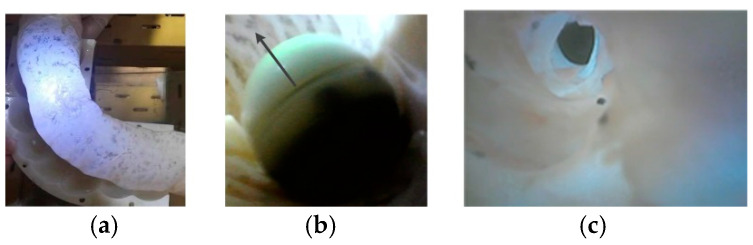
Posture detection application under porcine conditions.

**Figure 20:**
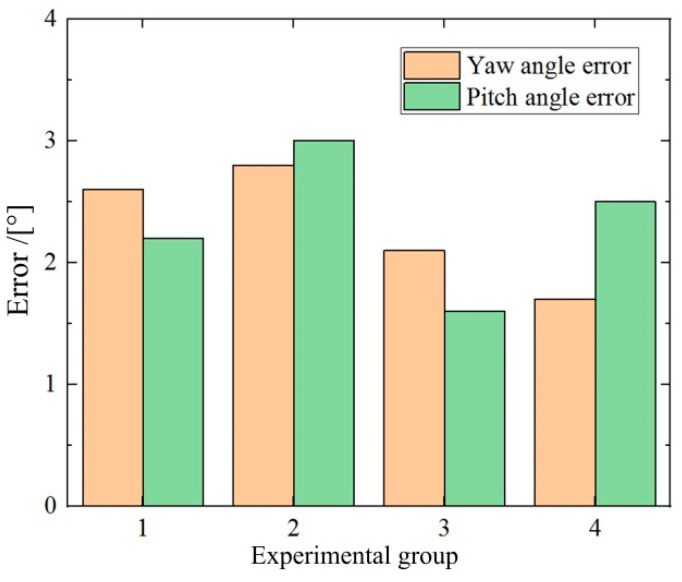
Experimental results of posture detection application.

To strengthen the claim of clinical applicability, proposed posture detection is applied in a porcine colon environment so that testing conditions more closely resemble in vivo scenarios, as shown in Figure 19. The DHCR is placed inside the porcine colon, and the DHCR movement is recorded by external and internal cameras. The actual value of the DHCR posture moving in the porcine colon is more challenging to measure. Navigation performance serves as an indirect validation metric, as the system calculates navigation direction from detected posture. For curved intestines, the dark zone is an important feature region. The navigation orientation from its mass center is derived based on using the improved OTSU algorithm. Different navigation orientations are set up to carry out the experiments, and the results are shown in Figure 20. The results show that the average error between the calculated and the actual values is within 2.3°. This error is superimposed by the errors of the posture detection method and the dark zone mass-center extraction method. The average error of the dark zone mass-center extraction method is about 1.3°. It is inferred that the posture detection error is about 1°. The marginally higher errors observed in biological tissue versus simulations likely resulted from real tissue deformations affecting DHCR motion and increased image matching complexity in vivo. The above experiments verify the feasibility of the proposed posture detection method.

## 6. Conclusions

The innovation of this paper is a posture detection method based on the ORB-AEKF algorithm for the magnetic-actuated DHCR. The method achieves posture calibration and adjustment based on tracking effects, combines GI tract image information acquired by the vision system with the ORB-based feature recognition and matching algorithm, and ultimately realizes the optimal estimation of the DHCR axial posture by using AEKF. This solution only requires a visual sensor, eliminating the need for additional inertial or magnetic sensors that would occupy the DHCR's internal space, which is conducive to further miniaturization. In addition, this method effectively fuses the dynamic response characteristics and visual observation information to enhance the DHCR posture estimation. The average detection errors of the yaw angle and pitch angle are 0.76° and 0.31° in simulated GI conditions. This method is applied with good results in porcine colon navigation experiments, providing an effective sensing feedback scheme for realizing closed-loop control of DHCR.

## Figures and Tables

**Table 1:** Posture detection calculation parameters.

Parameter	Value
Magnetic flux density *B*_0_	6 mT
Angular velocity *ω*	16π rad/s
Scale factor *ϑ*	0.8
Process noise covariance matrix ***Q***	diag(0.001, 0.001, 0.001, 0.001)
Observation noise covariance matrix ***R***	[5;5]

**Table 2:** Error statistics of DHCR posture detection methods.

Detection Method	Statistical Information	Yaw Angle	Pitch Angle
Posture detection based on ORB	error maximum value	4.87°	6.62°
	error mean value	1.67°	2.44°
	error variance	1.95	3.56
Posture detection based on ORB-EKF	error maximum value	4.79°	1.14°
	error mean value	1.53°	0.40°
	error variance	1.57	0.13
Posture detection based on ORB-AEKF	error maximum value	1.55°	0.76°
	error mean value	0.76°	0.31°
	error variance	0.16	0.07

## Data Availability

The data presented in this study are available on request from the corresponding author.

## Abbreviations

The following abbreviations are used in this manuscript:

**Abbreviation**	**Clarification**
GI	Gastrointestinal
MACR	Magnetic-actuated capsule robot
SURMF	Spatial universal rotating magnetic field
EKF	Extended Kalman filtering
HMI	Human–machine interaction
DHCR	Dual hemisphere capsule robot
ORB	Oriented FAST and rotated BRIEF
TOSHC	Three-axis orthogonal square Helmholtz coils
AEKF	Adaptive extended Kalman filtering

## Appendix A

The dynamic model is established based on the Lagrange equation, which can be described as follows:

(A1) $$\frac{d}{dt}\left(\frac{\partial L}{\partial {\dot{q}}_{i}}\right)-\frac{\partial L}{\partial q_{i}}=Q_{i}$$

where *q_i_* is the generalized coordinate; *L* is the Lagrangian, which can be expressed in terms of kinetic energy *T_L_* without considering the change in potential energy; and *Q_i_* is the generalized torque in the generalized coordinate system.

The DHCR axis posture can be expressed in the coordinate system using Euler angles (*α* and *β*), which are used as generalized coordinates *q_i_*.

The Lagrangian *L*, the sum of the kinetic energies of the two hemispheres, can be expressed as follows:

(A2) $$L=\frac{1}{2}{\omega_{a}}^{T}\left(\begin{array}{ccc} J_{a} & 0 & 0\\ 0 & J_{e} & 0\\ 0 & 0 & J_{e} \end{array}\right)\omega_{a}+\frac{1}{2}{\omega_{p}}^{T}\left(\begin{array}{ccc} J_{p} & 0 & 0\\ 0 & J_{e} & 0\\ 0 & 0 & J_{e} \end{array}\right)\omega_{p}$$

where *J_a_* and *J_p_* are the polar moments of inertia of the active and passive hemispheres, respectively, and *J*_e_ is the equatorial moment of inertia of the whole DHCR; and ***ω****_a_* and ***ω****_p_* are the angular velocities of the active and passive hemispheres, respectively.

When the DHCR operates in passive mode, it is mainly subjected to magnetic torque and GI contact resistance torque. Thus, generalized torque *Q_i_* includes magnetic torque *T_i_* and resistance torque *M_fi_* in the generalized coordinate system, expressed as follows:

(A3) $$Q_{i}=T_{i}+M_{fi}$$

To save the length of this paper, the items of the above equations are explained concerning to the coordinate system established in the literature [11], and the specific derivation process is shown in the literature [11].

## Appendix B

Quaternions are expressed as follows:

(A4) $$p=p_{0}+p_{1}i+p_{2}j+p_{3}k$$

where *p_i_* satisfies the following relation:

(A5) $${p_{0}}^{2}+{p_{1}}^{2}+{p_{2}}^{2}+{p_{3}}^{2}=1$$

The rotation matrix ***R****_I_* expressed through quaternions is as follows:

(A6) $$R_{I}=\left(\begin{array}{ccc} 1-2({p_{2}}^{2}+{p_{3}}^{2}) & 2(p_{1}p_{2}+p_{0}p_{3}) & 2(p_{1}p_{3}+p_{0}p_{2})\\ 2(p_{1}p_{2}-p_{0}p_{3}) & 1-2({p_{1}}^{2}+{p_{3}}^{2}) & 2(p_{2}p_{3}+p_{0}p_{1})\\ 2(p_{1}p_{3}+p_{0}p_{2}) & 2(p_{2}p_{3}+p_{0}p_{1}) & 1-2({p_{1}}^{2}+{p_{2}}^{2}) \end{array}\right)$$

The system state vector is composed of the rotation quaternion, and can be expressed as follows.

According to the derivation of Equation (A5), and the quaternion expression for the angular velocity ***ω****_p_*, the Euler kinematics equations in quaternion form can be solved:

(A7) $$\begin{array}{l} {\dot{p}}_{0}=\frac{1}{2}(-\omega_{x}p_{1}-\omega_{y}p_{2}-\omega_{z}p_{3}),{\dot{p}}_{1}=\frac{1}{2}(\omega_{x}p_{0}-\omega_{y}p_{3}+\omega_{z}p_{2}),\\ {\dot{p}}_{2}=\frac{1}{2}(\omega_{x}p_{3}+\omega_{y}p_{0}-\omega_{z}p_{1}),{\dot{p}}_{3}=\frac{1}{2}(-\omega_{x}p_{2}+\omega_{y}p_{1}+\omega_{z}p_{0}) \end{array}$$

It can also be expressed as follows:

(A8) $$\begin{array}{c} {\dot{X}}_{t}=\frac{1}{2}\Omega (\omega )X_{t}\\ \Omega (\omega )=\left(\begin{array}{cccc} 0 & -\omega_{x} & -\omega_{y} & -\omega_{z}\\ \omega_{x} & 0 & \omega_{z} & -\omega_{y}\\ \omega_{y} & -\omega_{z} & 0 & \omega_{x}\\ \omega_{z} & \omega_{y} & -\omega_{x} & 0 \end{array}\right) \end{array}$$

where the average rotational angular velocities *ω*_x_, *ω*_y_, and *ω*_z_ can be calculated from the dynamics in Equation (10) of the DHCR.

## Appendix C

### Appendix C.1. Prediction Phase

Assuming that the filter output, i.e., the a posteriori state estimate of the DHCR system at moment *k* − 1 is $\hat{X}(k-1|k-1)$, the a priori state estimate of the system at moment *k* is obtained as follows:

(A9) $$\hat{X}(k|k-1)=f[\hat{X}(k-1|k-1)]=[I_{4}+\frac{1}{2}\Omega (\omega )\Delta k]\hat{X}(k-1|k-1)$$

This equation is the prediction equation for the state of the system.

Similarly, the prediction equation for the observation of the system is expressed as follows:

(A10) $$\hat{Z}(k|k-1)=h[\hat{X}(k|k-1)]$$

Assuming that ***Q***(*k* − 1) is the process noise covariance matrix computed at moment *k* − 1, the system state prediction error variance matrix can then be expressed as follows:

(A11) $$P(k|k-1)=F(k|k-1)P(k-1|k-1)F^{T}(k|k-1)+Q(k-1)$$

where ***F***(*k*|*k* − 1) is the transfer matrix, obtained from the Jacobi matrix of the function *f* in Equation (9).

### Appendix C.2. Updating Phase

Assuming that ***R***(*k*) is the observation noise covariance matrix at moment *k*, the Kalman gain ***K***(*k*) at the current moment based on Equation (A11) is calculated as follows:

(A12) $$K(k)=P(k|k-1)H^{T}(k){\left[H(k)P(k|k-1)H^{T}(k)+R(k)\right]}^{-1}$$

where ***H***(*k*) is the observation matrix, obtained from the Jacobi matrix of the function *h* in Equation (16).

The priori estimate of the prediction is corrected by the Kalman gain, and the posteriori estimate is given by the following:

(A13) $$\hat{X}(k|k)=\hat{X}(k|k-1)+K(k)(Z(k)-\hat{Z}(k|k-1))$$

Then, the state error covariance matrix is updated as follows:

(A14) P(k|k)=[I−K(k)H(k)]P(k|k−1)

### Appendix C.3. Adaptive Covariance Matching

Define the new interest (observation residual) at moment *k* as the error between the actual value and the observed value, expressed as follows:

(A15) $$e(k)=Z(k)-\hat{Z}(k|k-1)$$

The actual new interest covariance matrix ***C****_e_*(*k*), is the following:

(A16) $$C_{e}(k)=\frac{1}{N}\sum_{i=1}^{N}e(k-i)e^{T}(k-i)$$

where *N* is the value of the sliding window.

The theoretical new interest covariance matrix ***C***(*k*) is the following:

(A17) $$C(k)=H(k)P(k|k-1)H^{T}(k)+R(k)$$

When ***C***(*k*) > ***C****_e_*(*k*), R(k) should be decreased; when ***C***(*k*) < ***C****_e_*(*k*), ***R***(*k*) should be increased. When ***R***(*k*) is larger than the actual noise distribution, it will cause the filter to diverge. Thus, ***R***(*k*) should be kept constant.

Kalman gain ***K***(*k*) by introducing a scaling factor in adaptive variance matching methods, can be expressed as follows:

(A18) $$K(k)=P(k|k-1)H^{T}(k){\left[H(k)P(k|k-1)H^{T}(k)+\alpha_{k}R(k)\right]}^{-1}$$

where *λ* is the scale factor, which takes the value of the following:

(A19) $$\lambda =\max(1,\frac{trace[C_{e}(k)]}{trace[C(k)]})$$

Generally, ***Q***(*k*) and ***R***(*k*) need to be inversely adjusted. Based on the above method, the system state prediction error variance matrix is updated with the following formula:

(A20) $$P(k|k-1)=F(k|k-1)P(k-1|k-1)F^{T}(k|k-1)+\frac{\vartheta }{\lambda }Q(k-1)$$

where *ϑ* is the scale factor, which is selected empirically.
